# Koch’s Reply to His Critics

**Published:** 1883-05-20

**Authors:** 


### KOCH'S REPLY TO HIS CRITICS.

In a recent communication to the Deutsche Medicine Wochenschrift, Dr. Robert Koch has published an elaborate review of the various criticisms made against his experiments and conclusions regarding the tubercle bacillus.

He premises by saying that investigators have somewhat lost sight of the chief point which he made, viz: that tuberculosis must be a parasitic infection because he caused it by inoculating the isolated parasites. He refers to the fact that in nearly all instances the bacilli have been found in phthisical patients and in no others. The few failures he attributes to lack of diligence in examination, the bacilli being sometimes few in number, or to want of skill in microscopic technique.

He then takes up the various criticisms and answers them in detail. We observe that it is only among Americans and Germans that he finds persons who have ventured to oppose his views.

Dr. Ephraim Cutter's opinion that Koch's discovery is not new, and that his bacilli are only the "babies" of Salisbury's *mycoderma aceti* is stated without comment.

Dr. Rollin R. Gregg "appears to have considered," says Koch, "that microscopical investigations would be superfluous for the establishment of his views."

Schmidt is commended for his honest desire to find the bacillus, but is advised that it would have been better if he had had the patience to wait till he had obtained good colors and learned how to use them. before announcing fat-crystals as bacilli.

Dr. Formad, of Philadelphia, is complimented as a more skillful microscopist. Still he has not, Koch thinks, yet learned to distinguish the bacilli of tuberculosis. He is further accused of being a prejudiced observer, having certain preconceived views regarding the lymphatic system of scrofulous animals. Finally, Koch states that Dr. Formad cannot give authoritative evidence upon the subject of tuberculosis until he has learned to find the bacilli with certainty, and until he has made himself familiar with the literature of tuberculous inoculations, especially of those of Cohnheim and Salomonsen, Hansell, Schuchardt, Baumgarten, and Damsch, and until he has become sufficientiy expert in experimental technique not to let his animals inoculated with wood, glass, and metal, die of tuberculosis.

Turning then to his German critics, Koch says: "If one thinks that German medicines cannot bring forth such blossoms of tubercle-bacilli literature as America, he is mistaken."

Beneke, who discovered, as he thought, bacilli in the ethereal extracts of the blood of healthy men, really found, says Koch, fat-crystals, like Schmidt's

Cramer announced that by using Ehrlich's coloring method he had found bacilli in the stools of twenty healthy persons. Koch cites contrary results obtained by Gaffky, and states that Cramer's bacilli were not identified with those of tuberculosis.

Balogh found in the Berlin mud, after a rain, bacilli like those of tuberculosis. Koch, from examinations of his own, contradicts Balogh, and denies any value to the inoculation experiments made by that investigator.

Schottelius produced anatomical tuberculosis in dogs by causing them to inhale masses of finely pulverized non-tuberculosis matter. Koch states that the anatomical appearance is not the criterion of what is tuberculous matter, and that Schottelius' experiments are completely contradicted by those of Bertheau and Wigart. Koch also argues against the view of Schottelius that bovine and human tuberculosis are not identical.

Detweiler has tried to show that the bacilli of tuberculosis are accompaniments, not causes of phthisis, because their inoculation is always followed by acute miliary tuberculosis, not by the pulmonary phthisis seen in man. Koch thinks he would change this view if he had a better knowledge of the pathology of tuberculosis.

Koch finally reviews Spina's recently published criticism, which has excited much attention, because this critic alone had repeated Koch's cultivations and inoculations.

Koch says that "Spina's microscopic technique is almost entirely different from that employed to-day in the study of bacteria." He speaks of Spina's "mistreatment of the bacilli with coloring methods," compares his work with that of Schmidt, and believes that all the new conclusions of Spina as regards staining are valueless. Spina's cultivation and inoculation experiments are also characterized as imperfect and ill-conducted, and as being but "characatures" of Koch.

Koch's reply shows how exacting and careful all experimenters must be in order to test fairly the problem he claims to have solved. It shows also Koch's great confidence in the fact that he alone so far has carefully, accurately, and impartially studied and settled the question.—*N. Y. Med. Record.*

